# An American Text-Book of Physiology

**Published:** 1901-03

**Authors:** 


An American Text-Book of Physiology. By Ten of the Leading Physiologists of America. Edited by William H. Howell, Ph. D., M. D., Professor of Physiology in the Johns Hopkins University, Baltimore. Octavo, 1052 pages. Illustrated. Second edition, revised. Volume I. Philadelphia: W. B. Saunders & Co. 1900. [Price, cloth, $6 00 net; sheep or half Morocco, $7.00 net.

The American Text-Book of Physiology first appeared in 1896. It took rank at once as a standard if, indeed, not the leading American work on this subject. The second edition has been most carefully revised. Not only has new matter been incorporated with the text wherever such changes are called for, but statements and theories which time has proven wrong or impossible have been eliminated. Among additions to the first volume is to be noted an especially interesting section upon the modern view of the processes of osmosis and diffusion. Whoever uses the work will appreciate the advantage of two easily handled volumes over one so large as to be cumbersome.

